# Supplementary material for: Intranasal delivery of shRNA to knockdown the 5HT-2A receptor enhances memory and alleviates anxiety
Source: Transl Psychiatry. 2024 Mar 20;14:154. doi: 10.1038/s41398-024-02879-y (PMC10954635; doi:10.1038/s41398-024-02879-y)
Supplement: Supplementary file 1 — Supplementary Information [file 41398_2024_2879_MOESM1_ESM.docx]

**Supplementary Information**

**Figure 1**

**
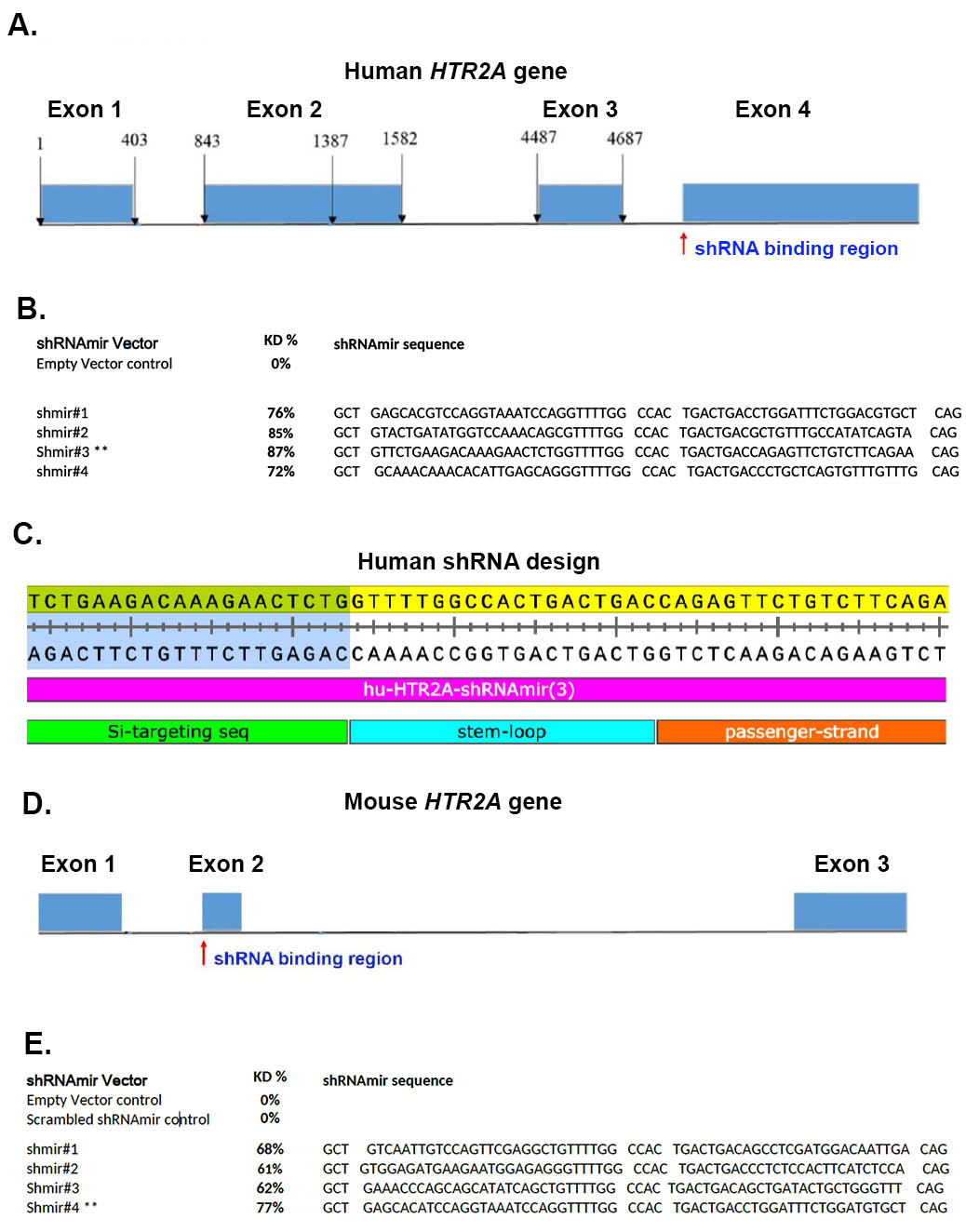
**

**Supplementary Fig. 1. Targeting strategy to knock down the human and mouse *HTR2A* gene using shRNA.** **(A)**: The *HTR2A* human gene located on chromosome 13q14-21 spans 66 kilobases and consists of 4 exons. At least 2 major isoforms are generated from alternative splicing between exons 1-2. Therefore, we chose to construct a shRNA sequence targeting the beginning of exon 4, which would lead to degradation of the largest coding exon and potentially downregulation of the two major isoforms of the human *HTR2A* gene. **(B)**: Four different shRNA constructs were validated and screened *in vitro*. Shmir#3, which produced an 87% knockdown was selected to be used for viral production.

**(C)**: The design of the human shRNA consists of three parts: 1) The target sequence shown (shaded sequence) and depicted in green; 2) The stem-loop region depicted in light blue; 3) The passenger strand sequence shown in orange. Details on the construction of this shRNA can be found in the methods section. **(D)**: The *HTR2A* mouse gene encodes a single protein-coding transcript, *Htr2a-201*, located on chromosome 14. A similar strategy as to constructing the human shRNA was employed to design the mouse version. In this case, the target sequence was designed to bind at the beginning of exon 2. Silencing of exon 2 would prevent the 5HT-2A receptor protein from being produced. **(E)**: Four different shRNAs were tested for potential knockdown *in vitro*. Shmir#4 was chosen for viral production following demonstration of a 77% knockdown with no knockdown observed either with the empty vector control or a scrambled shRNA control.

**Figure 2**

**
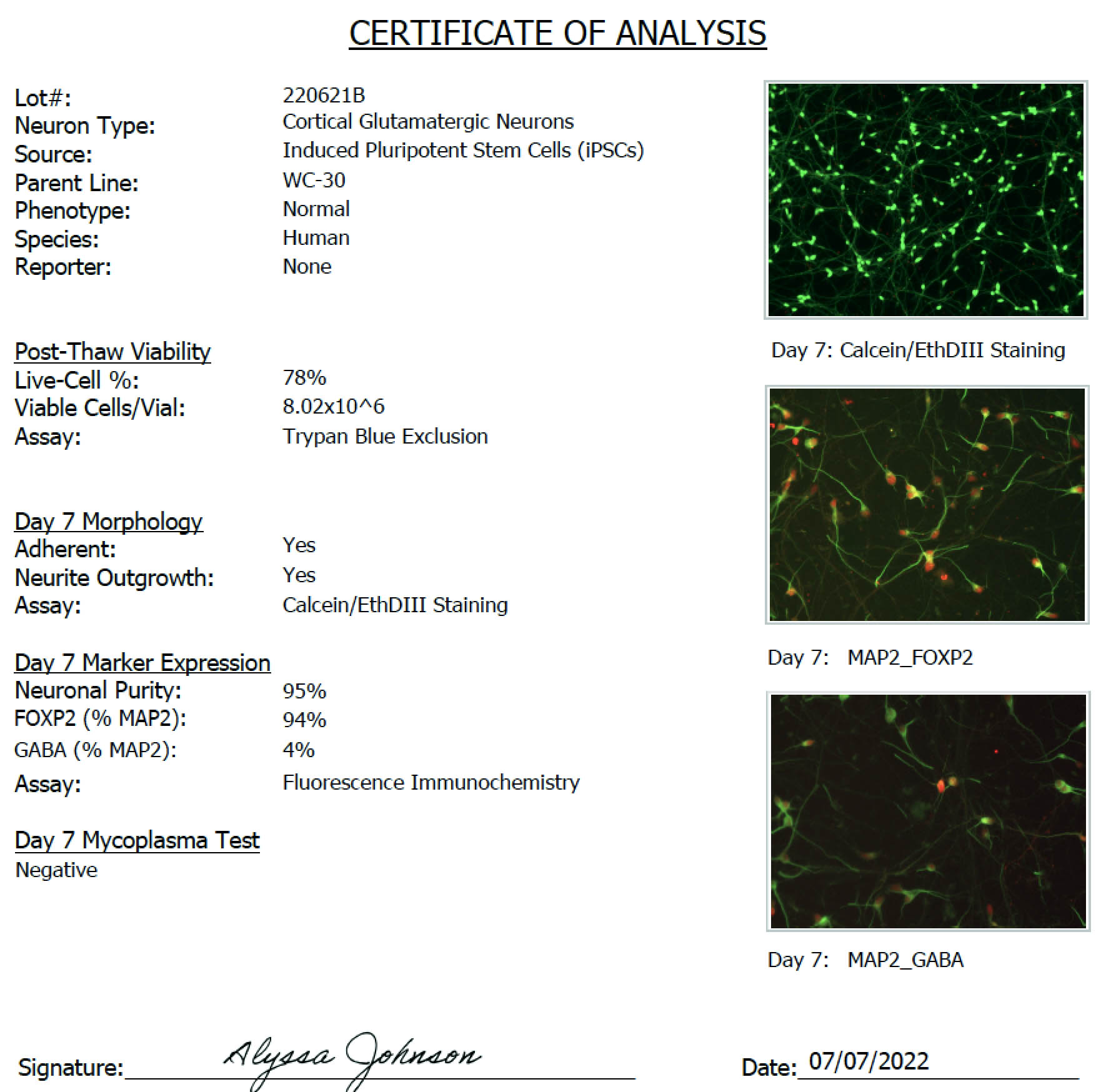
**

**Supplementary Fig. 2. Certificate of analysis for human iPSC cortical glutamatergic neurons.** This certificate confirms the purity and identity of cortical glutamatergic neurons derived from induced pluripotent stem cells (iPSCs) of the WC-30 line, assessed at 7 days *in vitro*. Immunocytochemistry was performed to identify neuronal purity and subtype specification. Additionally, post-thaw viability was confirmed at 78%, with 8.02x10^6 viable cells per vial as determined by trypan blue exclusion. Cells exhibited adherence and neurite outgrowth, as shown by Calcein/EthDIII staining. The culture was negative for Mycoplasma contamination, ensuring the integrity of the study. Images depict representative fields of neurons stained for the respective markers. The upper right image shows Calcein (green)/EthDIII (red) staining, indicating live/dead cells. The bottom left image shows MAP2-positive (green) neurons, a subset of which are FOXP2-positive (red), and the bottom right image shows MAP2-positive neurons with a minority also staining for GABA (red).

**Table 1: Seeding medium for culturing human iPSC differentiated neurons**

| Component | Stock Conc. | Final Conc. | 1 Plate volume |
| --- | --- | --- | --- |
| DMEM/F12 Medium | 1X | 0.5X | 9.5 mL |
| Neurobasal Medium | 1X | 0.5X | 9.5 mL |
| B27 Supplement | 50X | 1X | 400 µL |
| N2 Supplement | 100X | 1X | 200 µL |
| GlutaMAX | 200 mM | 0.5 mM | 50 µL |
| BDNF | 10 µg/mL | 10 ng/mL | 20 µL |
| GDNF | 10 µg/mL | 10 ng/mL | 20 µL |
| TGF-β1 | 1 µg/mL | 1 ng/mL | 20 µL |
| Geltrex | 15 mg/mL | 15 µg/mL | 200 µL |
| Glut Neuron Seeding Supplement | 1000X | 1X | 20 µL |
| Supplement K | 1000X | 0.5X | 10 µL |

Table 2: Day 4 medium for culturing human iPSC differentiated neurons

| Component | Stock Conc. | Final Conc. | 1 Plate volume |
| --- | --- | --- | --- |
| DMEM/F12 Medium | 1X | 0.25X | 4.8 mL |
| Neurobasal Medium | 1X | 0.25X | 4.8 mL |
| BrainPhys Medium | 1X | 0.5X | 9.6 µL |
| B27 Supplement | 50X | 1X | 400 µL |
| N2 Supplement | 100X | 1X | 200 µL |
| GlutaMAX | 200 mM | 0.5 mM | 50 µL |
| BDNF | 10 µg/mL | 10 ng/mL | 20 µL |
| GDNF | 10 µg/mL | 10 ng/mL | 20 µL |
| TGF-β1 | 1 µg/mL | 1 ng/mL | 20 µL |
| Day 4 Supplement | 1000X | 1X | 20 µL |
| Supplement K | 1000X | 0.5X | 10 µL |

Table 3: Maintenance media for culturing human iPSC differentiated neurons

| Component | Stock Conc. | Final Conc. | 1 Plate volume |
| --- | --- | --- | --- |
| BrainPhys Medium | 1X | 0.5X | 19.3 mL |
| B27 Supplement | 50X | 1X | 400 µL |
| N2 Supplement | 100X | 1X | 200 µL |
| GlutaMAX | 200 mM | 0.5 mM | 50 µL |
| BDNF | 10 µg/mL | 10 ng/mL | 20 µL |
| GDNF | 10 µg/mL | 10 ng/mL | 20 µL |
| TGF-β1 | 1 µg/mL | 1 ng/mL | 20 µL |
